# Conventional vs. endoscopic-assisted curettage of benign bone tumours. An experimental study

**DOI:** 10.1186/s13018-024-04859-w

**Published:** 2024-07-05

**Authors:** Maria Anna Smolle, Lukas Jud, Fabrice André Scheurer, Armando Hoch, Jakob Ackermann, Benjamin Fritz, Daniel Andreas Müller

**Affiliations:** 1 Department of Orthopedic Surgery, Balgrist University Hospital, University of Zurich, Forchstrasse 340, Zürich, 8008 Switzerland; 2 Department of Orthopaedics and Trauma, Medical University of Graz, Auenbruggerplatz 5, Graz, 8036 Austria; 3 Department of Radiology, Balgrist University Hospital, University of Zurich, Forchstrasse 340, Zürich, 8008 Switzerland

**Keywords:** Curettage, Benign bone tumour, Endoscopy, Orthopaedic oncology

## Abstract

**Background:**

This experimental study aimed at directly comparing conventional and endoscopic-assisted curettage towards (1) amount of residual tumour tissue (RTT) and (2) differences between techniques regarding surgical time and surgeons’ experience level.

**Methods:**

Three orthopaedic surgeons (trainee, consultant, senior consultant) performed both conventional (4x each) and endoscopic-assisted curettages (4x each) on specifically prepared cortical-soft cancellous femur and tibia sawbone models. “Tumours” consisted of radio-opaque polyurethane-based foam injected into prepared holes. Pre- and postinterventional CT-scans were carried out and RTT assessed on CT-scans. For statistical analyses, percentage of RTT in relation to total lesion’s volume was used. T-tests, Wilcoxon rank-sum tests, and Kruskal-Wallis tests were applied to assess differences between surgeons and surgical techniques regarding RTT and timing.

**Results:**

Median overall RTT was 1% (IQR 1 – 4%). Endoscopic-assisted curettage was associated with lower amount of RTT (median, 1%, IQR 0 − 5%) compared to conventional curettage (median, 4%, IQR 0 − 15%, *p* = 0.024). Mean surgical time was prolonged with endoscopic-assisted (9.2 ± 2.9 min) versus conventional curettage (5.9 ± 2.0 min; *p* = 0.004). No significant difference in RTT amount (*p* = 0.571) or curetting time (*p* = 0.251) depending on surgeons’ experience level was found.

**Conclusions:**

Endoscopic-assisted curettage appears superior to conventional curettage regarding complete tissue removal, yet at expenses of prolonged curetting time. In clinical practice, this procedure may be reserved for cases at high risk of recurrence (e.g. anatomy, histology).

**Supplementary Information:**

The online version contains supplementary material available at 10.1186/s13018-024-04859-w.

## Background

Intralesional curettage is commonly used for treatment of benign bone tumours including enchondroma, chondroblastoma, aneurysmal bone cyst, and giant cell tumour of bone [1–5]. Preservation of original bone stock and low morbidity can be seen advantageous of this surgical procedure [6, 7]. Yet, curettage of benign bone tumours naturally harbours the risk of remaining tumour cells within the cavity, potentially leading to local recurrence [8–11]. Curettage can be challenging and may necessitate large cortical fenestration to achieve a good visualization of the bony cavity. At the same time, the invasiveness may lead to long-term morbidity. Adjuvants as phenol, liquid nitrogen and hydrogen peroxide as well as bone cement with its thermal reaction during consolidation all aim at reducing recurrence rates by leading to necrosis of remnant tumour cells. Yet, the most important factor to minimise recurrence remains meticulous curettage [12]. Consequently, the bone window developed to reach – and visually inspect – the entire tumour cavity has to be sufficiently large [13, 14]. 

Endoscopic curettage through a small cortical hole with arthroscopic devices constitutes an alternative to open curettage, as described for several benign bone tumours [15–19]. However, the smaller the cortical window, the more limited the curettes’ cruising radius will be. A hybrid method is the combination of limited open curettage through a bone window by additional visual inspection with an endoscope, thus combining the potential advantages of both procedures, i.e. adequate exposure and visibility [9, 13, 15, 16]. 

A study directly comparing the efficacy of conventional vs. endoscopic-assisted curettage regarding completeness of tissue removal has not been carried out thus far.

The aim of this experimental study therefore was to (1) compare the completeness of curettage with conventional in comparison to endoscopic-assisted curettage, and to (2) assess potential differences between the two techniques regarding curetting time and surgeons’ experience level.

Consequently, the findings of our study may fill the knowledge gap on the additive value of endoscopy to conventional curettage in terms of complete tumour tissue removal.

## Methods

### Materials

**Fig. 1:**
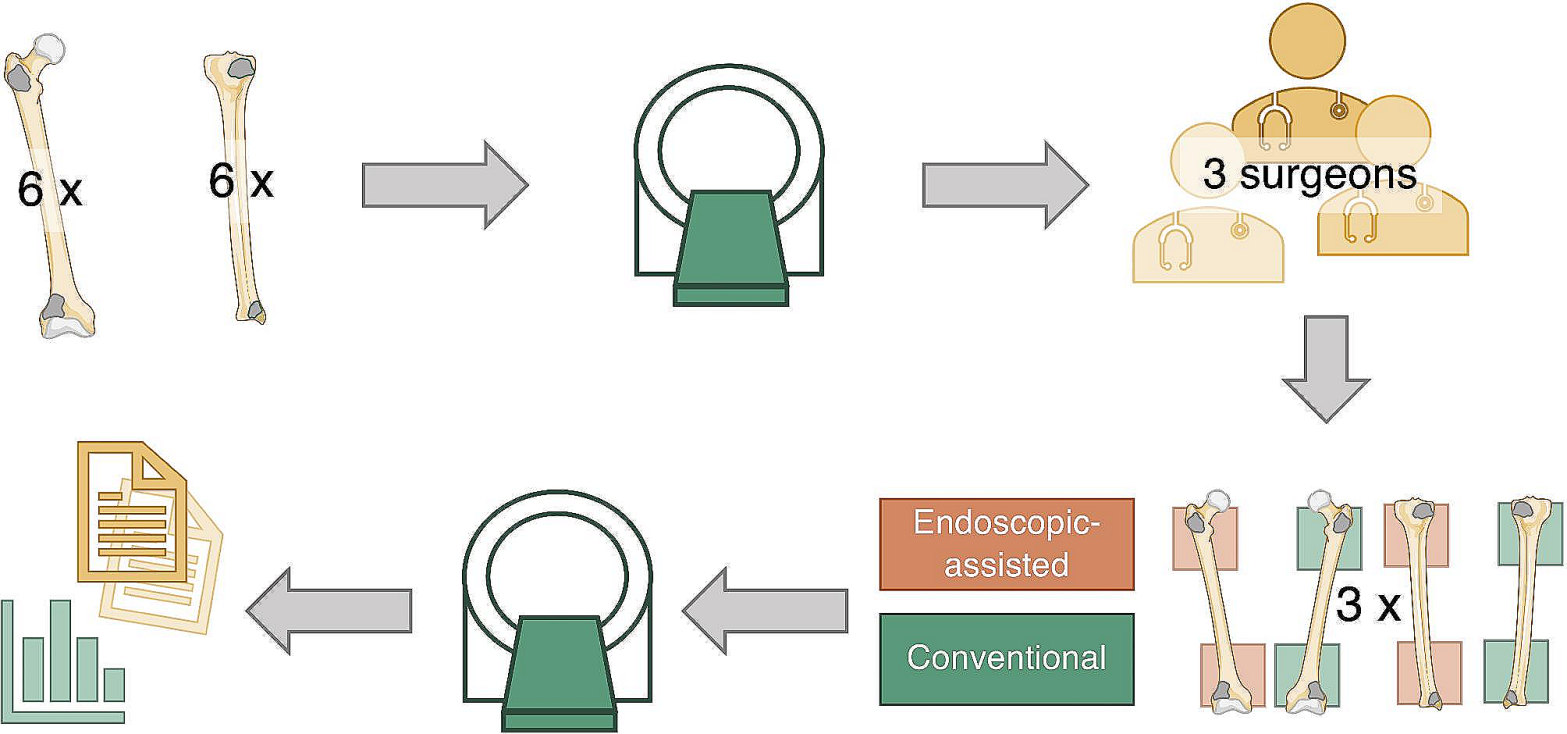
Graphical visualisation of the study workflow. Tumour cavities were prepared at the proximal and distal metaphyses of six femoral and tibial sawbones. Subsequently, CT-scans of the prepared sawbones were carried out. Thereafter, each surgeon performed curettages on two femoral and two tibial sawbones, once with the conventional (green) and once with the endoscopic-assisted technique (orange). Following intervention, all sawbones again underwent CT-scans. Ultimately, image analysis of pre- and postinterventional CT-scans was carried out

For the experimental setup (Fig. 1), twelve cortical soft-cancellous sawbones (*SYNBONE® AG, Zizers, Switzerland*) were used (6 femur: *SYNBONE®*, product no. 2350.9; 6 tibia: *SYNBONE®*, product no. LSH1385.9).

### Methods

At the proximal and distal metaphyseal area of each sawbone, holes were carved via a standardised cortical window (15 mm x 15 mm) to mimic later tumour cavity. This resulted in altogether 24 holes (intertrochanteric region, bone window ventral [*n* = 6]; distal femur, bone window lateral [*n* = 6]; proximal tibial condyle, bone window medial [*n* = 6]; distal tibia, bone window cranial to medial malleolus [*n* = 6]). Following sealing of the medullary canal with a cement restrictor to avoid leakage, holes were filled with a contrast-medium enriched polyurethane-based foam. Although holes were prepared in a standardised manner, the resulting lesions’ volumes slightly differed between anatomical locations (**Additional File 1**).

Three orthopaedic surgeons with different experience levels (trainee, consultant [< 5 years of experience], senior consultant [> 5 years of experience]) carried out the curettages. Every surgeon performed 8 curettages, four times each with the conventional and endoscopic-assisted technique. No minimum curetting time was defined, yet maximum curetting time was limited to 15 min. As soon as surgeons were certain to have removed the entire foam, the experiment was stopped, and the resulting curetting time documented.

**Fig. 2:**
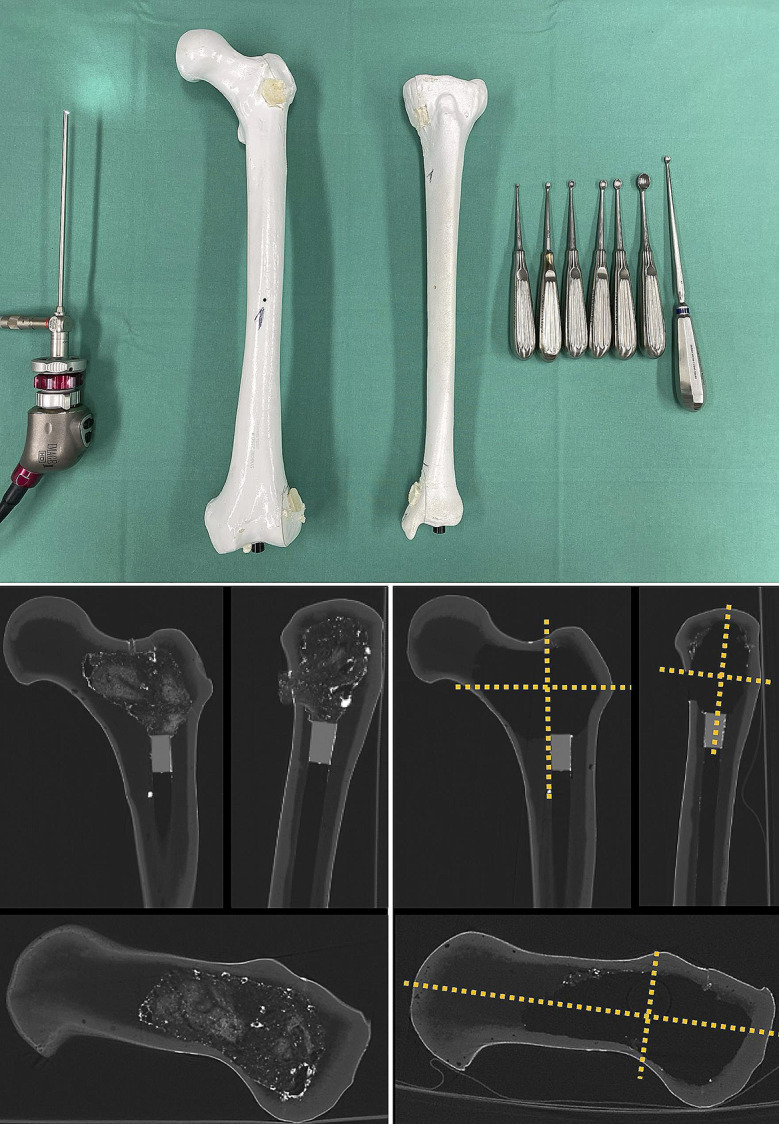
Experimental built-up. (Top) Picture of the experimental built-up showing endoscope (left), prepared femoral and tibial sawbone (middle) and curettes used (right). (Bottom, left) Pre-interventional CT scan of a proximal femoral sawbone in 3 planes (coronal, sagittal, axial) depicting the cement restrictor to seal the medullary canal and the contrast-enhanced foam used to mimic the lesion. (Bottom, right) CT-scan in 3 planes (coronal, sagittal, axial) of the same proximal femoral sawbone following curettage, with contrast-enhanced foam still visible at the lesion’s periphery. Orange lines define segments used for radiological assessment of RTT.

For the conventional curettage, spoons and curettes of varying sizes and angles commonly used in clinical practice, were provided (Fig. 2, **top**). For the endoscopic-assisted technique, a commercially available endoscope was used (*IMAGE 1 HD, 1.9 mm 30° HOPKINS II Autoclavable, Karl Storz, Tuttlingen, Germany*; Fig. 2, **top**). The endoscope was subsequently inserted into the cavity to view potentially remnant foam. Apart from the endoscope, no further endoscopic or arthroscopic devices were used.

After preparation of the sawbones, computer-assisted tomography (CT) scans (*Siemens Naeotom Alpha; slice thickness spiral 0.20 mm / reconstruction 2 mm; kilovoltage [kV] 120*) were carried out to enable measurements of the initial lesions’ volume (Fig. 2, **bottom left**). Volume of lesions was estimated as expansion in craniocaudal x mediolateral x anteroposterior plane. Following curettage, CT scans were repeated with the same protocol (Fig. 2, **bottom right**), as described previously [20]. 

A senior consultant musculoskeletal radiologist (B.F.) assessed all pre- and post-interventional CT-images. For standardised image analysis, each lesion was divided into 8 segments. These segments were defined by three orthogonal planes in horizontal and vertical orientation along the midlines of the “tumour” cavities. In lesions of the distal femur and proximal as well as distal tibia, these planes were oriented along the axis of the diaphysis. In proximal femoral lesions, the craniocaudal and mediolateral planes were oriented along the femoral neck axis, and the horizontal plane along the axis of the diaphysis (Fig. 2, **bottom right**).

Consequently, 192 segments were analysed in total (48 segments per tumour location). Volume of residual tumour tissue (RTT) was documented within each of these segments (expansion in craniocaudal x mediolateral x anteroposterior plane). By summarising RTTs per segment, the total RTT was calculated.

### Statistical analysis

Means are provided with corresponding standard deviations (SDs) and medians with interquartile ranges (IQRs) and ranges. Distribution of variables was tested with Shapiro-Wilk test. T-tests, Wilcoxon rank-sum tests and Kruskal-Wallis tests were applied to assess differences in RTT and curetting time between surgeries and surgeons, as appropriate. Correlations between curetting time and amount of RTT were assessed with Pearson’s correlation coefficient. For better comparability, percental RTT relative to the total lesion’s volume was used for statistical analyses. All statistical analyses were carried out with SPSS for Mac (*Version 23.0, SPSS Inc., Chicago, IL, US*) and Stata Version 16.1 for Mac (*StataCorp, College Station, TX, US*). A p-value of < 05 was considered statistically significant.

## Results

### General findings

**Fig. 3:**
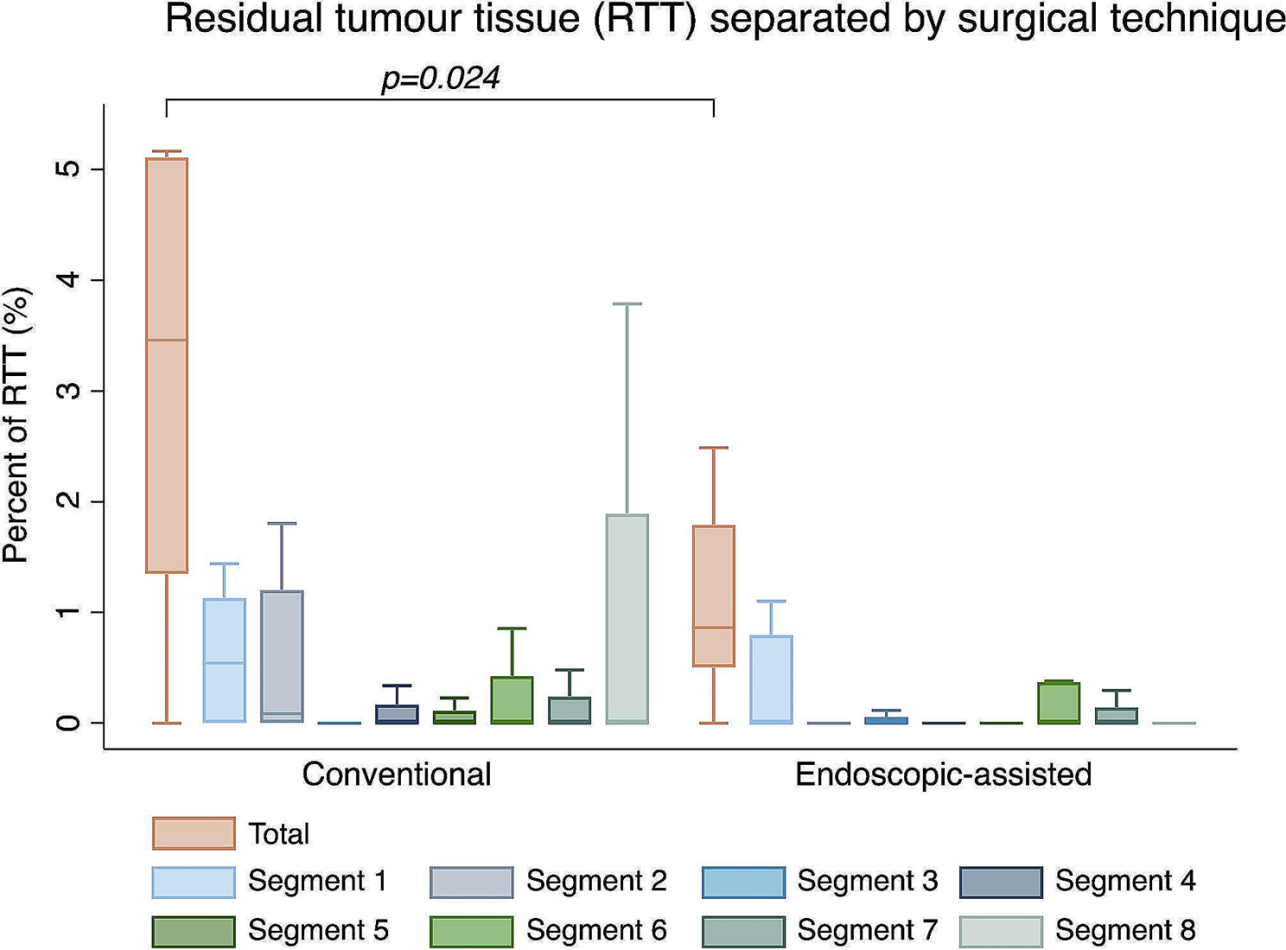
Overall percental residual tumour tissue (RTT) as well as RTT per segment analysed, separated by surgical technique. Green and blue bars show median percental RTT per segment with 25th and 75th percentile, and orange bars the sum of RTTs within each segment with 25th and 75th percentile. Whiskers denote lower and upper adjacent values*. P-value based on Wilcoxon rank-sum test. *adjacent values defined as 25th or 75th percentile + 1.5 x interquartile range

Median lesions’ volume was equivalent to 72,453 mm^3^ (IQR: 45,294–94,494 mm^3^; range: 22,792–140,400 mm^3^) and significantly differed between locations (*p* = 0.005; **Additional File 1**). Over all segments, median RTT volume amounted to 1257 mm^3^ (IQR: 630–2976 mm^3^; range: 264–16,100 mm^3^). Median percental RTT relative to each lesion’s total volume, was equivalent to 1.3% (IQR: 0.7 – 4.1%; range: 0.0 – 15.0%). Figure 3 depicts percental RTT within each segment and over all segments combined, separated by surgical technique.

### Completeness of curettage depending on technique

Endoscopic-assisted curettage was associated with a significantly lower median amount of RTT (0.9%; IQR: 0.5 – 1.8%; range 0.0 – 5.2%) compared to conventional curettage (3.5%; IQR: 1.3 – 5.1%; range 0.0 − 15.0%; *p* = 0.024; Table 1; Fig. 3). When analysing differences in RTTs of each segment depending on surgical technique separately, the only significant difference was present for segment 2 (Table 1). Furthermore, no significant difference between the three surgeons regarding overall percental RTT was found (*p* = 0.571).

**Table 1:** Median percentage of residual tumour tissue (RTT) per segment relative to lesion’s total volume as analysed on CT-scans, separated by surgical technique

Segments	Overall	Endoscopic-assisted technique	Conventional technique	*p*-value
Segment 1				
*Median [IQR]* *Range* *Lesions with RTT*	0.2 [0.0–1.0] 0.0–3.9 [12]	0.0 [0.0–0.8] 0.0–3.9 [7]	0.5 [0.0–1.1] 0.0–3.4 [5]	0.517
**Segment 2**				
*Median [IQR]* *Range* *Lesions with RTT*	0.0 [0.0–0.2] 0.0–1.8 [7]	0.5 [0.0–0.0] 0.0–0.5 [1]	0.0 [0.0–1.2] 0.0–1.8 [6]	0.026*
**Segment 3**				
*Median [IQR]* *Range* *Lesions with RTT*	0.0 [0.0–0.0] 0.0–0.4 [4]	0.0 [0.0–0.1] 0.0–0.4 [3]	0.0 [0.0–0.0] 0.0–0.2 [1]	0.307
**Segment 4**				
*Median [IQR]* *Range* *Lesions with RTT*	0.0 [0.0–0.0] 0.0–0.7 [4]	0.0 [0.0–0.0] 0.0–0.5 [1]	0.0 [0.0–0.2] 0.0–0.7 [3]	0.266
**Segment 5**				
*Median [IQR]* *Range* *Lesions with RTT*	0.0 [0.0–0.0] 0.0–2.4 [4]	0.0 [0.0–0.0] 0.0–0.2 [1]	0.0 [0.0–0.1] 0.0–2.4 [3]	0.230
**Segment 6**				
*Median [IQR]* *Range* *Lesions with RTT*	0.0 [0.0–0.4] 0.0–2.5 [7]	0.0 [0.0–0.4] 0.0–2.5 [4]	0.0 [0.0–0.4] 0.0–1.9 [3]	0.774
**Segment 7**				
*Median [IQR]* *Range* *Lesions with RTT*	0.0 [0.0–0.1] 0.0–6.3 [6]	0.0 [0.0–0.1] 0.0–1.1 [3]	0.0 [0.0–0.2] 0.0–6.3 [3]	0.791
**Segment 8**				
*Median [IQR]* *Range* *Lesions with RTT*	0.0 [0.0–0.0] 0.0–8.2 [5]	0.0 [0.0–0.0] 0.0–0.2 [2]	0.0 [0.0–1.9] 0.0–8.2 [3]	0.464
**Overall**				
*Median [IQR]* *Range*	**1.3 [0.7–4.1]** 0.0–15.0*	**0.9 [0.5–1.8]** 0.0–5.2*	**3.5 [1.3–5.1]** 0.0–15.0*	0.024*

Medians, IQRs and ranges are given as percental RTT; Numbers in brackets indicate lesions positive for RTT; IQR – interquartile range; RTT – residual tumour tissue

*asterisks indicate significant results; p-values based on Wilcoxon rank-sum tests

### Relevance of curetting time & surgeon’s experience level

**Fig. 4:**
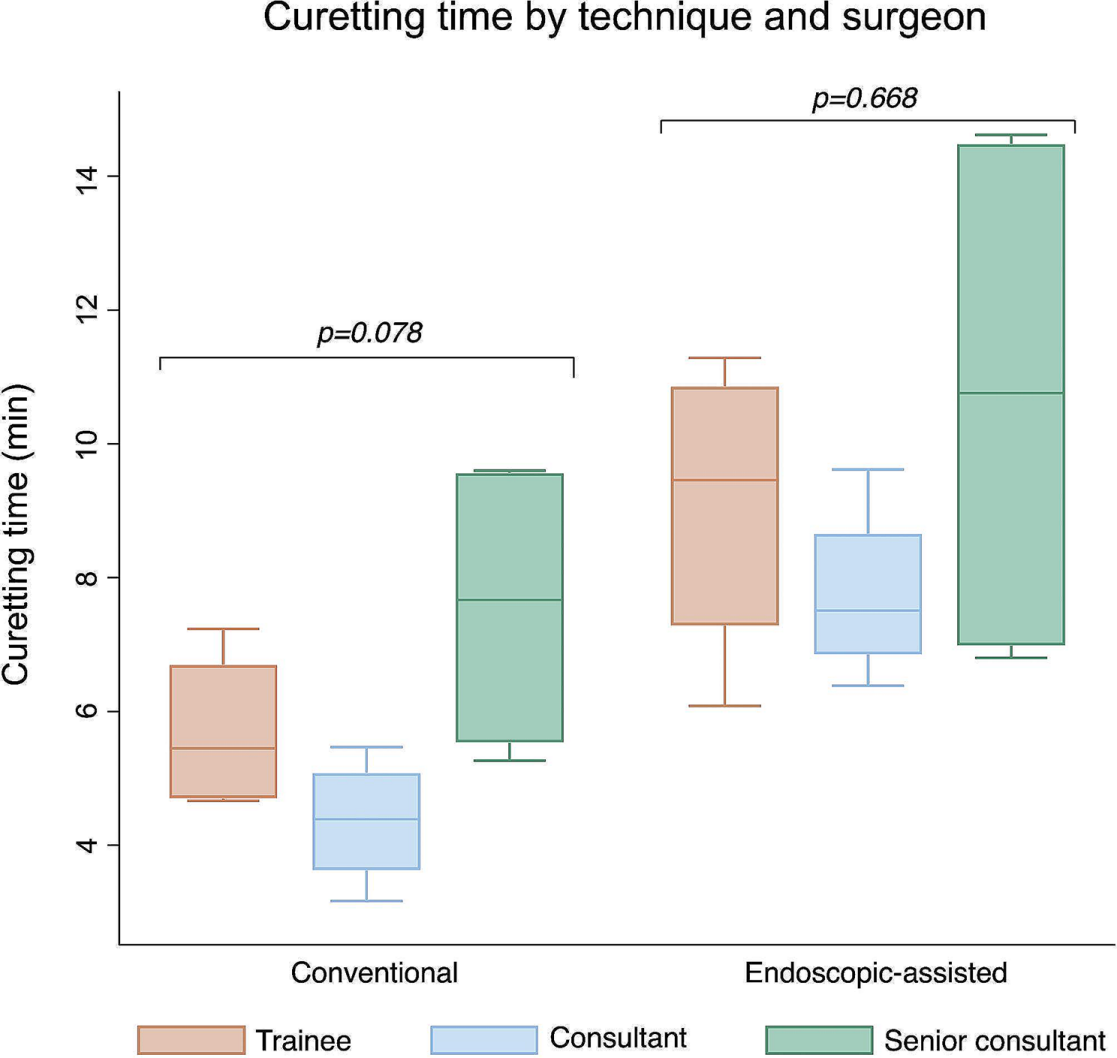
Difference in curetting time between surgeons depending on technique. Bars depict median curetting time with 25th and 75th percentile. Whiskers denote lower and upper adjacent values*. P-values based on Kruskal-Wallis test. *adjacent values defined *as 25th or 75th percentile + 1.5 x interquartile range*

With a mean of 9.2 ± 2.0 min, endoscopic-assisted curettage took significantly longer than the conventional one (5.9 ± 2.1 min; *p* = 0.004). Overall mean curetting time was 7.5 ± 3.0 min. No significant correlation between curetting time and amount of RTT was found (Pearson’s *r*=-0.174; *p* = 0.450). Curetting time was comparable between all three surgeons, regardless of their experience level (*p* = 0.251). In addition, no significant difference in curetting time between surgeons depending on technique was evident (conventional *p* = 0.078; endoscopic-assisted *p* = 0.668; Fig. 4).

## Discussion

Conventional curettage is the mainstay of surgical treatment for various benign bone tumours and tumour-like lesions. Owing to the risk of residual tumour tissue, the addition of endoscopy to curettage has been proposed in clinical practice to enhance visualisation.

Here, we discovered that endoscopic-assisted curettage is associated with a significantly lower percentage of residual tumour tissue as compared with conventional curettage. At the same time, curetting time is longer with endoscopic-assisted curettage. Further, no difference in amount of RTT or curetting time depending in surgeons’ experience level is present.

This experimental study has some limitations. First, “tumour” lesions were prepared at four different anatomical locations, resulting in varying lesions’ sizes. Therefore, each surgeon carried out curettages at every location with the two techniques, and percental RTT rather than total RTT volume was used for statistical analysis. Furthermore, volumes of the artificially created “tumours” and volumetric differences depending on anatomical location align with in-vivo findings [21, 22]. At the same time, the standardised cortical windows of 15 × 15 mm herein implemented did not account for varying tumour dimensions that would in clinical practice require proportionately sized approaches [13, 14, 21]. Second, the overall number of lesions curetted was small (*n* = 24), wherefore subgroup analyses beyond stratification between surgeons and surgical techniques were not carried out. Third, a potential apprehension bias [23] has to be considered given that surgeons were aware of the study’s hypothesis. This may serve as an additional explanation why no significant difference in terms of curetting time was found despite the surgeons’ varying experience levels. Further, we were only able to directly compare curetting times rather than the potential entire surgical procedure including access to bone through soft tissues, and subsequent wound closure. In addition, the experimental set-up did not allow to replicate potential difficulties of an in-vivo curettage with bleeding and debris eventually compromising visibility.

Advantages of conventional curettage over an extended open approach include its low invasiveness, small cortical bone window, and fast postoperative recovery [6, 7]. On the other hand, limited intraoperative visualisation, risk for intraoperative fracture due to aggressive curettage through a small cortical window and potential of remnant tumour cells leading to later recurrence constitute disadvantages [17, 18]. Correspondingly, a recent systematic literature review [12], reported an overall recurrence rate of 12.5% following conventional curettage of benign bone tumours. These recurrences frequently require further surgeries, thus affecting patients’ quality of life [24]. 

To reduce risk of remnant tumour cells, some authors have reported that the addition of endoscopy to conventional curettage enables better visualisation of the tumour cavity [9, 14–16, 19]. For example, *Aiba et al.* successfully applied endoscopic-assisted curettage for the treatment of aneurysmal [16] as well as simple bone cysts [15]. Similarly, endoscopic-assisted curettage has been used to treat unicameral bone cysts of the calcaneus [13] and femur [14]. With this technique, recurrence rates between 0% [14], 10% [16] and 18.9% [15] have been achieved, being lower than the ones observed following conventional curettage [10, 11, 16]. However, none of these studies directly compared the two techniques in terms of performance and timing. Consequently, we performed an experimental study directly comparing conventional and endoscopic-assisted curettage regarding RTT and curetting time.

We discovered that the endoscopic-assisted technique is associated with significantly less RTT within the lesions’ cavity (median, 0.9%) compared to conventional curettage (median, 3.5%). In parallel, an increase in curetting time was observed with endoscopic-assisted (mean, 9.2 min) versus conventional curettage (mean, 5.9 min). Owing to the experimental setup of the study, no direct comparison to findings made in clinical practice are possible. Intriguingly, though, *in-vivo* surgical times appear shorter with the endoscopic-assisted curettage (median 45 to 108 min [15, 16, 19]) compared to the conventional technique (mean 115 to 171 min [4, 5]). An explanation for these discrepant findings could be the surgical approach, which is more limited with the endoscopic-assisted technique [16], as well as subsequent easier wound closure, two factors that were not addressed within the setup of this experimental study.

Interestingly, we did not observe any difference in RTT or curetting time between the three surgeons, all with varying levels of experience. Intriguingly, long-lasting clinical experience appeared to even prolong curetting time, whilst amount of RTT remained unaffected. Although only hypothesis-generating, one explanation for these findings may be the raised awareness of well-experienced orthopaedic physicians towards the considerable effects recurrences have on patients’ outcomes, whilst younger, less experienced physicians may be less biased towards this clinical observation. Furthermore, none of the assessors are highly experienced in arthroscopic surgery, serving as another potential explanation for varying curetting times. Regardless of surgeons’ experience levels, though, endoscopic-assisted curettage improves visualisation and eventually reduces risk for remnant tumour cells, wherefore this technique may also be employed in training of junior surgeons. In clinical practice, endoscopy may be added to conventional curettage in tumours known for a high recurrence risk (e.g. giant cell tumour of bone), or in case of critical anatomical locations (e.g. pelvic girdle, femoral head, proximal tibia, vicinity to joints/growth plates) [25, 26]. 

## Conclusions

Our findings confirm the hypothesis that endoscopic-assisted curettage is superior to conventional curettage in terms of complete tissue removal. The higher precision of this technique is at the expenses of prolonged curetting time, though. Transferred to clinical practice, endoscopy may be added to curettage especially in cases at increased risk for RTT due to histopathology and/or anatomical location. Whilst this likely enhances completeness of curettage, the question whether surgical time will be prolonged remains to be answered.

### Electronic supplementary material

Below is the link to the electronic supplementary material.

Supplementary Material 1

## Data Availability

The datasets used and/or analysed during the current study are available from the corresponding author on reasonable request.

## Abbreviations

CT: Computer-assisted tomography
IQR: Interquartile range
RTT: Residual tumour tissue
SD: Standard deviation
