# Correction: Conventional vs. endoscopic-assisted curettage of benign bone tumours. An experimental study

**DOI:** 10.1186/s13018-024-04930-6

**Published:** 2024-07-25

**Authors:** Maria Anna Smolle, Lukas Jud, Fabrice André Scheurer, Armando Hoch, Jakob Ackermann, Benjamin Fritz, Daniel Andreas Müller

**Affiliations:** 1 Department of Orthopedic Surgery, Balgrist University Hospital, University of Zurich, Forchstrasse 340, Zürich, 8008 Switzerland; 2 Department of Orthopaedics and Trauma, Medical University of Graz, Auenbruggerplatz 5, Graz, 8036 Austria; 3 Department of Radiology, Balgrist University Hospital, University of Zurich, Forchstrasse 340, Zürich, 8008 Switzerland

**Correction: Journal of Orthopaedic Surgery and Research (2024) 19:392**

10.1186/s13018-024-04859-w

In this article, Maria Anna Smolle and Lukas Jud should have been denoted as an equally contributing authors.

The original article has been corrected.

