# Supplementary material for: Conventional vs. endoscopic-assisted curettage of benign bone tumours. An experimental study
Source: J Orthop Surg Res. 2024 Jul 5;19:392. doi: 10.1186/s13018-024-04859-w (PMC11225110; doi:10.1186/s13018-024-04859-w)
Supplement: Supplementary file 1 — Supplementary Material 1 [file 13018_2024_4859_MOESM1_ESM.docx]

**Additional File 1**

**Manuscript Title: Conventional vs. endoscopic-assisted curettage of benign bone tumours. An experimental study.**

Running title: Conventional vs. endoscopic curettage.

**Additional File 1**. Median lesions’ volume in mm^3^ depending on location.

*Note the statistically significant difference between the locations (p=0.005); IQR – interquartile range*

| **Location** | **Median volume (in mm^3^)** |
| --- | --- |
| **Proximal femur** |  |
| *Median [IQR]*  *Range* | 78430 [66240 – 115920]  63000 – 140400 |
| Distal femur |  |
| *Median [IQR]*  *Range* | 84740 [65664 – 107604]  60588 – 111628 |
| Proximal tibia |  |
| *Median [IQR]*  *Range* | 79689 [73226 – 92988]  69654 – 96000 |
| Distal tibia |  |
| *Median [IQR]*  *Range* | 24669 [23166 – 27300]  22792 – 30000 |
